# Adamantinoma-like ewing sarcoma arising in the pancreatic tail: a case report of a rare entity and review of the literature

**DOI:** 10.1186/s13000-023-01374-0

**Published:** 2023-07-31

**Authors:** Zhe Wang, Xiaobo Wen, Yingchun Zhang, Xinke Zhang

**Affiliations:** 1 Department of Pathology, The Eighth Affiliated Hospital, Sun Yat-sen University, Shenzhen, Guangdong 518033 China; 2 Department of Pathology, Sun Yat-sen University Cancer Center, No. 651, Dongfeng Road East, Guangzhou, China

**Keywords:** ALES, Pancreatic tail, Differential diagnosis

## Abstract

ALES is a rare subtype that demonstrates the EWSR1-FLI1 translocation characteristic of ES and demonstrates complex epithelial differentiation including diffuse cytokeratin and p40 expression. It has predominantly recognized in the head and neck and is common in middle-aged population. This case is the first case of ALES reported in the pancreatic tail, sharing some morphological characteristics with ALES in the head and neck, including monotonous cytology, infiltrative growth pattern, and complex epithelioid differentiation, but ALES in the head and neck often has high-grade histological features (e.g., necrosis, high mitotic rate, etc.), and sudden keratinization can also occur, but these features were not reflected in this primary pancreatic tail ALES. Although ALES arising in the pancreatic tail and in the head and neck sites share the immunohistochemical and molecular profile, our case can provide new ideas in differential diagnosis of ALES arising in pancreatic tail and promote increased recognition and understanding of ALES.

## Introduction

In the latest version of the WHO classification of soft tissue and bone tumors, Ewing sarcoma (ES) is defined as a malignant small blue round cell tumor that occurs in bone and soft tissue, with common genetic characteristics, including fusions of FET (EWSR1/FUS/TAF15, etc.) gene family members with transcription factor ETS (FLI1/ERG, etc.) family members. The most commonly EWSR1-FLI1 fusion accounts for approximately 85% of all confirmed ES cases [1]. Almost all ES cases have diffused and strong positive membrane expression of CD99. This antibody is very sensitive but not specific. FLI1 can be expressed in ES with EWSR1-FLI1 gene fusion. In recent years, NKX2.2 has been proved to be a more specific immune marker for diagnosis of ES [2]. Therefore, the combination of CD99, NKX2.2, and FLI1 helps to distinguish ES from other histologically similar tumors [3]. In addition, ES may express non-specific markers, such as synaptophysin, NSE, Leu-7, etc. due to neuroectodermal differentiation. Adamantinoma-like Ewing sarcoma (ALES) is a rare and somewhat controversial variant of ES that was initially reported by van Haelst et al. in 1975, accounting for about 5% of ES. ALES predominantly occurred in the head and neck sites [4]. The most characteristic morphological manifestations of ALES are fibrous tissue septation, tumor cell nests showing an epithelial differentiation, palisade-like arrangement and rosette-like structures. Immunohistochemistry usually shows diffuse membrane staining of CD99, significant nuclear expression of FLI1 and NKX2.2, and in particular expression of epithelial markers including CK, P40 and CK5/6. Molecular testing revealed an EWSR1-FLI1 gene fusion traditionally regarded as pathognomonic for a diagnosis of ES, which can also specifically confirm the diagnosis of ALES [5]. Currently reported head and neck ALES can pose a particularly challenging differential diagnosis with squamous cell carcinomas of poor differentiation and basaloid carcinomas [6]. This report aims to firstly present a characteristic clinical, pathologic, immunophenotypic, and molecular features of the primary pancreatic tail ALES, highlighting differential diagnosis which is different from that occurring in the head and neck, and facilitating better understanding of ALES.

## Case presentation

**Fig. 1:**
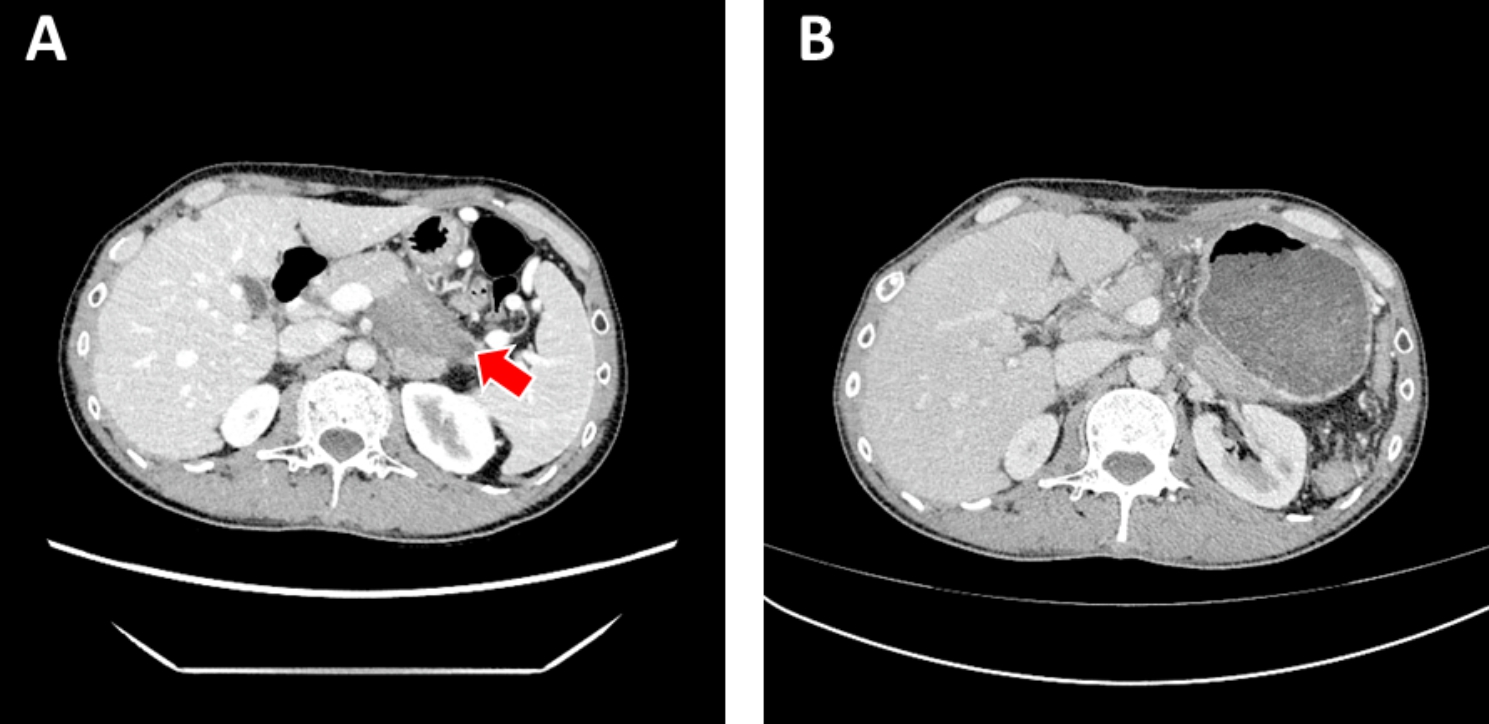
CT scan of the abdomen Hypodense mass (arrow) in the body and tail of the pancreas at diagnosis **(A)**; The absence of body and tail of the pancreas, left adrenal gland and spleen after operation, and a small amount of effusion in the original operation area is basically absorbed after 3 courses of chemotherapy with CAV/IE regimen **(B)**

The patient was a 43-year-old male who had dull-aching epigastric pain without apparent reason for one month. On radiological evaluation: The pancreatic body and tail lesion with a size of about 65*36mm was highly suspected diagnosis of pancreatic cancer (Fig. 1A). The boundary with the left prerenal fascia was unclear, and the splenic artery and vein were invaded, accompanied by the formation of splenic vein emboli. Lateral branch nodules of the left adrenal gland suggested the possibility of metastases. Tumor marker detection showed serum alpha-fetoprotein (AFP) level (1.86ng/ml), carbohydrate antigen 125 (CA125) level (11.30U/ml), carbohydrate antigen 19 − 9 (CA19-9) level (10.9U/ml), and carcinoembryonic antigen (CEA) level (3.23ng/ml), which were no obvious abnormalities. Subsequently, laparoscopic exploration + radical surgery of pancreatic cancer + left adrenalectomy was performed in our hospital.

**Fig. 2:**
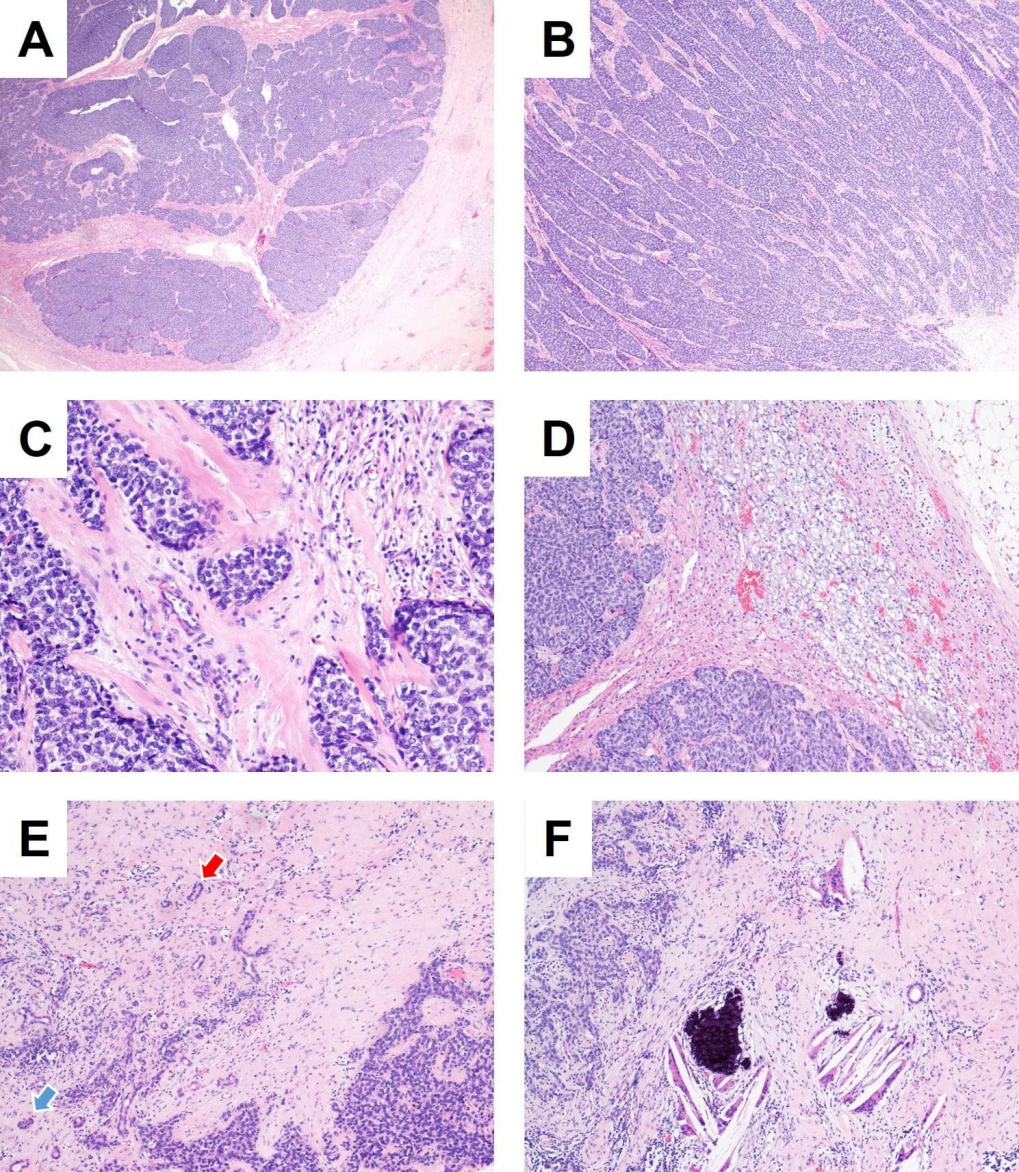
Adamantinoma-like Ewing Sarcoma arising in the pancreatic tail of a 43 year-old male. ALES consisted of lobules, trabeculae, or irregular nests of basaloid tumor cells (**A**, 2x; **B**, 4x) embedded in a prominent hyalinized or fibromyxoid stroma (**C**, 20x). It is highly infiltrative into adrenal tissue (**D**, 10x) and pancreatic parenchyma, shown as atrophic glands (blue arrow) and residual ductal structures (red arrow) (**E**, 10x). Cholesterol cleft, scattered giant cells and residual ductal structures were seen in the desmoplastic stroma around the tumor (**F**, 10x)

**Fig. 3:**
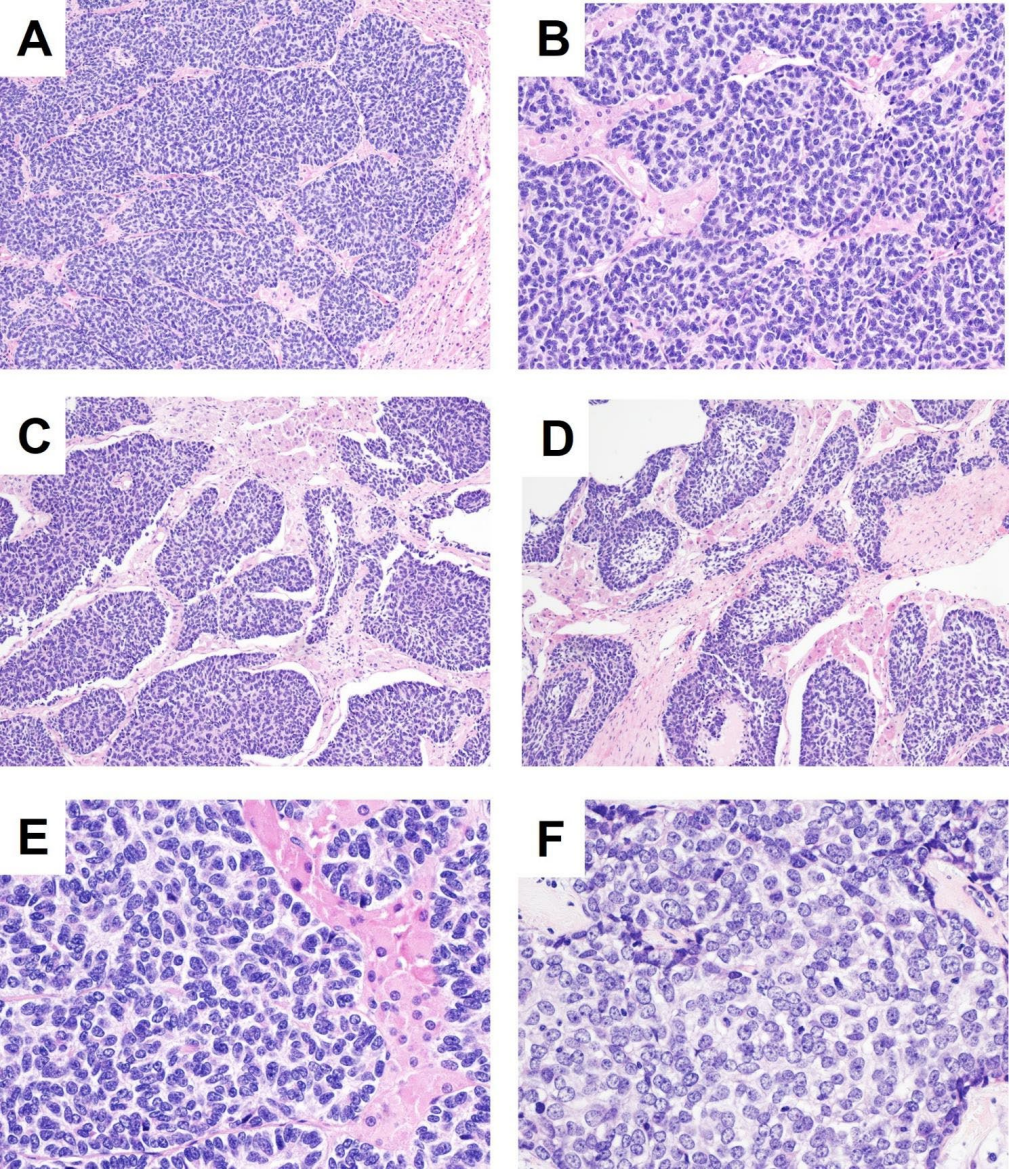
Tumor nests showed peripheral nuclear palisading (**A**, 10x), rosette structures (**B**, 20x), cleft formation (**C**, 10x), and epithelial differentiation like adamantinoma (**D**, 10x). Tumor cells were monotonous with minimal clear to basaloid cytoplasm, round to oval nuclei, vesicular chromatin, variably prominent micro nucleoli, and low mitotic activity (**E**, 40x; **F**, 40x)

Gross evaluation revealed a tumor in the tail of pancreas where is 2 cm away from the pancreatic stump, with a size of 65*50*40 mm, grayish white appearance, tough texture, and involving adrenal gland tissue. The histological morphology showed that the tumor cells were distributed in the form of infiltrative lobules, trabeculae, or irregular nests of basaloid cells embedded in a prominent hyalinized or fibromyxoid stroma (Fig. 2A and B C). While some tumor nests show peripheral nuclear palisading, rosette structures and cleft formation between tumor lobules and stroma (Fig. 3A and B C). The tumor nests in part showed epithelial differentiation similar to adamantinoma [7], which is arranged in peripheral palisading, and the loosely stellate reticular layer in the central area (Fig. 3D). Tumor cells were monotonous and uniform with minimal clear to eosinophilic cytoplasm, round to oval nuclei, finely granular or vesicular chromatin, variably prominent micro nucleoli, and low mitotic activity (Fig. 3E F). No areas of necrosis were found. Interestingly, the tumor-infiltrated pancreatic parenchyma (Fig. 2E), which showed atrophic glandular and residual ductal structures surrounded by desmoplastic stroma, with cholesterol cleft, scattered giant cells and calcification (Fig. 2F).

**Fig. 4:**
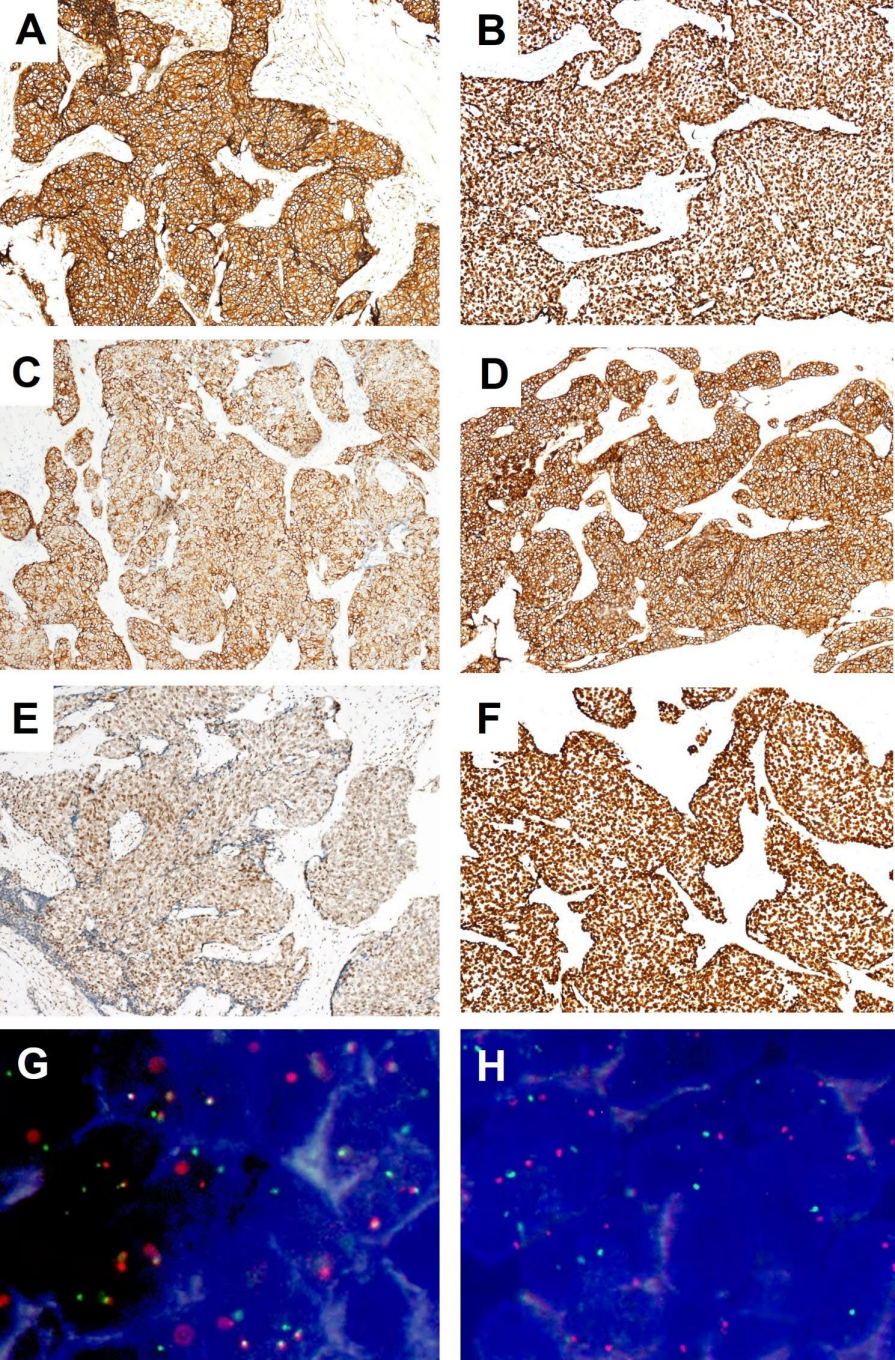
ALES was strongly and diffusely positive for cytokeratin AE1/AE3 (**A**, 10x), p40 (**B**, 10x), CK5/6 (**C**, 10x), membranous CD99 (**D**, 10x), and NKX2.2 (**F**, 10x), with varying degrees of nuclear positivity for FLI-1(**E**, 10x). ALES also demonstrated EWSR1-FLI1 translocations (**G**) without EWSRI-WT1 gene fusions (**H**) on fluorescent in-situ hybridization

Immunohistochemistry revealed tumor cells were diffusely positive for cytokeratin (AE1/AE3), P40, and CK5/6 (Fig. 4A and B C). In addition, neoplastic cells shared characteristics of conventional Ewing Sarcoma, with diffuse and strong membranous expression of CD99 (Fig. 4D), nuclear expression of NKX2.2 (Fig. 4F), with varying degree of nuclear positivity for FLI1 (Fig. 4E). Ki67 index was about 20%. Additionally, Cytokeratin 7 and 20, CD56, synaptophysin, chromogranin, SF1, α-inhibin, WT1, desmin, SMA, GATA3, Uroplakin II, CD10, PR, CD5, BerEP4, and NUT were negative in tumor cells, no loss of INI1 expression, β-catenin showed only membranous staining without nuclear accumulation (no shown).

Fluorescence in situ hybridization for both EWS gene break and the EWSR1-FLI1 gene fusion were positive (Fig. 4G), and EWSR1-WT1 translocation is negative (Fig. 4H). Based on the morphological, immunohistochemical, and molecular findings, a diagnosis of Adamantinoma-like Ewing Sarcoma (ALES) was rendered, and tumor cells infiltrated into pancreatic parenchyma and adrenal tissue (Fig. 2D).

It is worth noting that the present case showed aggressiveness, but was without high-grade morphological features, including overt necrosis and high mitotic activity, which are common in head and neck ALES. At the same time, a total of 30 lymph nodes in 7 groups removed were all negative for malignancy. After the operation, the patient was receiving a chemotherapy regimen which is the CAV plan (vincristine, doxorubicin and cyclophosphamide) alternating with the IE plan (ifosfamide and etoposide). At present, the patient has completed the chemotherapy of CAV/IE regimen for 3 times. The patient tolerates it well and remains disease-free by imaging 4 months after diagnosis (Fig. 1B).

## Discussion

Looking back at all the reports on ALES, almost all of them reported in the head and neck, including salivary glands, thyroid gland [8], nasal cavity and soft tissues of the head and neck [6]. Among these, ALES arising from the salivary glands is the most common. A latest literature summarizes 21 cases with salivary gland ALES [9]. Median age at diagnosis was 46 years, and ALES shows a male: female ratio of 1:1.6. Most tumors originated in the parotid gland, and skeletal and mesenteric metastases were described in a single patient. Surgery was performed in all reported cases and more than half of patients received adjuvant chemoradiotherapy. After a median follow-up of 13 months with ranging from 1 to 96 months, absence of local or systemic recurrence was documented in most cases. This suggests that most of ALES cases have the low tendency to local and/or metastatic dissemination, which is different from classic Ewing sarcoma (ES). Although the patient in our study was receiving a ES-specific chemotherapy regimen, tolerates it well and remains disease-free by imaging 4 months after diagnosis, the single death recorded in previous study was secondary to the postoperative ES-specific regimens [9]. Thus, it is necessary to recognize and diagnose this variant of ES from prognosis and treatment point of view. Owing to rarity of this entity, we need to collect more ALES cases to evaluate this difference of treatment and prognosis between ALES and classic ES. While our case is the first case of ALES reported in the pancreatic tail. Therefore, the differential diagnosis of current case will be different from that of ALES in head and neck sites.

Histological appearance is characterized by nests of small round blue cells embedded in the hyalinized or fibromyxoid stroma with an infiltrative growth. The leading differential diagnostic consideration in this case was desmoplastic small round cell tumor (DSRCT) [10]. DSRCT primarily affects children and young adults, and more commonly occurs in the abdominal cavity. It is well defined nests of highly malignant small round cells separated by desmoplastic stroma. Uniform cells with small hyperchromatic nuclei, inconspicuous nucleoli, scant cytoplasm, indistinct cytoplasmic borders and high mitotic rate. Rosette-like structures may be observed occasionally. Immunohistochemically, the tumor cells are immunoreactive for cytokeratins and desmin and show strong nuclear expression for WT1 (C terminus) [11] and harbor a distinctive EWSR1-WT1 fusion in the molecular pathology [12]. While in this case of ALES, the tumor cells did not express desmin and WT1 and was negative for diagnostic-specific EWSR1-WT1 translocation. With similar morphological features, including monotonous, scant cytoplasm, round to oval nuclei with fine, powdery chromatin, an extraskeletal undifferentiated round cell sarcoma with EWSR1-CREB3L1 fusion gene also need to be differentially diagnosis [13]. This definition is firstly reported in 2021, they describe this kind of undifferentiated round cell (“Ewing-like”) sarcoma. Although it is similar to our ALES morphologically, it does not have the immunophenotype of epithelial differentiation (negative for AE1/3, CK5/6, P63) and has EWSR1-CREB3L1 fusion rather than EWSR1-FLI1.

Since radiology description indicated that this case appears as hypoattenuating masses involving body and tail of the pancreas, tumors arising from the pancreas need to be ruled out. The morphologic appearance should firstly trigger consideration of pancreatic neuroendocrine tumors [14], especially well differentiated neuroendocrine tumor (WDNET) grade 3, which is more common in body and tail of the pancreas. The morphology of WDNET shows organoid architecture, such as solid nests and trabeculae, and small to medium cells with finely granular cytoplasm, round/oval nuclei, with salt and pepper (finely stippled) chromatin, no or inconspicuous nucleoli. These cells express neuroendocrine markers including CD56, synaptophysin and chromogranin A [15]. The current case shared similar morphological features with WDNET G3, but does not express neuroendocrine markers, which can basically rule out the diagnosis. In addition, pancreatic lesions with appearance of cholesterol cleft, scattered giant cells and calcification usually suggest a diagnosis of solid pseudopapillary tumor, solid areas of which may be comprised of uniform cells with rare mitotic Figures [16]. But, the morphological findings of the surgical gross specimens did not demonstrate variable admixture of solid and pseudopapillary areas. Also, immunohistochemical staining did not show nuclear β-catenin reactivity and was negative for CD10 and PR [17]. All these features did not support the diagnosis of solid pseudopapillary tumors. It is worth noting that we did the fine-needle aspiration (FNA) before the operation. Due to the limited biopsy tissue, findings initially showed hyperplastic fibrous tissue and residual pancreatic ductal structures under the microscope, no neoplastic components were found. This would explain the existence of cholesterol clefts and macrophages in the postoperative sample.

At the same time, imaging studies also suggest that there is a solid mass in adrenal gland, and the histomorphology also shows that there is multiple tumor nests infiltration in the adrenal gland, so the primary adrenal gland tumor also needs to be identified. Uniform small round blue cells distributed in nests and the formation of rosette-like structures can be seen. Therefore, the sex cord-stromal tumor arising in adrenal gland needs to be identified firstly, especially granulosa cell tumor [18]. This tumor is one of the adrenocortical tumors with sex cord-stromal differentiation, which is extremely rare. To date, only 6 cases of sex cord-stromal tumor in the adrenal gland have been documented, including 3 cases of granulosa cell tumor and 3 cases of leydig cell tumor [19]. All of them occurred in postmenopausal women. Histomorphologically, tumor cells are small, bland, oval cells with scant cytoplasm and uniform angulated and usually grooved nuclei (coffee bean), they could be arranged in various patterns, including diffuse, trabecular and corded, insular, microfollicular and macrofollicular. The immunohistochemical profiles are similar to those in the ovary or testis. Tumor cells are typically positive for α-inhibin, calretinin, SF1, Melan-A, CD56 and CD99, and negative for EMA and occasional expression of other keratin markers. However, in our case of ALES, the tumor cells demonstrated epithelial differentiation with diffuse CK and CK5/6 positivity, but was negative for α-inhibin, SF1, CD56, and WT1, which basically ruled out the diagnosis of primary adrenal granulosa cell tumor, and further ruled out the possibility of pancreas metastasis of adult testis granulosa cell tumor [20]. Additionally, malignant tumors arising of the adrenal gland are commonly adrenocortical carcinoma and pheochromocytoma. The histology of current case did not show the characteristic morphological features of these two diseases, and our case did not express the specific markers of the adrenal cortex (e.g., SF1) and medulla (e.g., CgA).

Aside from primary tumors, metastasis of surrounding epithelial tumors needs to be considered. According to morphological appearance, the first differential diagnosis is the metastasis of invasive urothelial carcinoma, especially nested urothelial carcinoma [21, 22]. It demonstrates bland, low grade nuclear features, may be accompanied by desmoplastic stroma and cleft formation around the tumor nest, and may appear focal squamous or glandular differentiation. ALES may overlap the morphology of nested urothelial carcinoma, but urothelial markers such as GATA3, UroII, CK7, CK20 cannot be detected in current case. It is well-known that almost all the case reports of ALES occur in the head and neck. Therefore, diseases which may easily lead to the misdiagnosis of head and neck ALES need to be considered to rule out pancreatic tail metastasis of occult head and neck tumors. Based on the histological appearance with a broad spectrum of tumors which show squamous or neuroendocrine differentiation, mimic tumors with squamous differentiation and consistent P40 positivity should be firstly excluded, including poorly differentiated squamous cell carcinoma [23] and NUT carcinoma [24]. Both of them show highly infiltrative growth but lacks diffuse CD99 positivity and exhibits more marked nuclear pleomorphism than ALES. In addition, NUT immunostaining is highly sensitive for NUT carcinoma and uniformly negative in ALES. As mentioned above, ALES in this case does not express any neuroendocrine markers (e.g., CD56, Syn, and CgA). Consequently, the metastasis of head and neck neuroendocrine tumors such as nasal neuroendocrine carcinoma [25], olfactory neuroblastoma [26], and Merkel cell carcinoma [27] can be excluded. In the head and neck, salivary gland tumors account for a large proportion, most of them have biphasic ductal and myoepithelial/basal cell populations. Our ALES presented tumor nests with monorous cells without biphasic differentiation. Besides P40, our ALES is negative for other myoepithelial markers, including SMA, and Calponin, and CD10 [28]. Finally, in addition to the morphological differences, this ALES did not express PAX-8, CD5, Ber-EP4 and loss of INI1 expression was not seen, further excluding the pancreas metastasis of intrathyroidal thyroid undifferentiated carcinoma [29], thymic carcinoma [30], skin basal cell carcinoma [31] and INI1-defient undifferentiated carcinoma [32].

In summary, we reported this case of primary pancreatic tail ALES firstly. It is invasive growth involving in solid organs including the pancreas and adrenal gland. As a rare tumor, ALES was usually demonstrated in the head and neck, and it has many potential diagnostic pitfalls when it is arising in the pancreatic tail. Therefore, it is important to recognize the morphological, immunohistochemical, and molecular genetic diagnostic criteria of ALES, so that an accurate diagnosis can be made. This report will provide new insights into the diagnosis of small round cell tumors in solid organs, and help facilitate the recognition of ALES and further insights into this rare tumor.

## Data Availability

Consent for publication was obtained from the patient. This consent was for the intervention, and the study derived from the sample for diagnosis, publications, or research.
